# Copula-based measures of asymmetry between the lower and upper tail probabilities

**DOI:** 10.1007/s00362-022-01297-w

**Published:** 2022-03-06

**Authors:** Shogo Kato, Toshinao Yoshiba, Shinto Eguchi

**Affiliations:** 1 Institute of Statistical Mathematics, 10-3 Midori-cho, Tachikawa, Tokyo 190-8562 Japan; 2 Graduate School of Management, Tokyo Metropolitan University, 18F 1-4-1 Marunouchi, Chiyoda-ku, Tokyo 100-0005 Japan

**Keywords:** Asymptotic theory, Bootstrap, Extreme value theory, Gaussian process, Stock daily return, 62E15, 62G32

## Abstract

We propose a copula-based measure of asymmetry between the lower and upper tail probabilities of bivariate distributions. The proposed measure has a simple form and possesses some desirable properties as a measure of asymmetry. The limit of the proposed measure as the index goes to the boundary of its domain can be expressed in a simple form under certain conditions on copulas. A sample analogue of the proposed measure for a sample from a copula is presented and its weak convergence to a Gaussian process is shown. Another sample analogue of the presented measure, which is based on a sample from a distribution on $\mathbb {R}^2$, is given. Simple methods for interval and region estimation are presented. A simulation study is carried out to investigate the performance of the proposed sample analogues and methods for interval estimation. As an example, the presented measure is applied to daily returns of S&P500 and Nikkei225. A trivariate extension of the proposed measure and its sample analogue are briefly discussed.

**Supplementary Information:**

The online version contains supplementary material available at 10.1007/s00362-022-01297-w.

## Introduction

In statistical analysis of multivariate data, it is often the case that data have complex dependence structure among variables. As a statistical tool for analyzing such data, copulas have gained their popularity in various academic fields, especially, finance, actuarial science and survival analysis (see, e.g., Joe (1997, 2014); Nelsen (2006); McNeil et al. (2015)).

A copula is a multivariate cumulative distribution function with uniform [0, 1] margins. The bivariate case of Sklar’s theorem states that, for a bivariate cumulative distribution function *F* with margins $F_1$ and $F_2$, there exists a copula *C* such that $F(x_1,x_2)=C(F_1(x_1),F_2(x_2))$. Hence a copula can be used as a model for dependence structure and is applicable for flexible modeling. Another important advantage of using copulas is that copulas are useful as measures of dependence. For example, the tail dependence coefficient is well-known as a measure of dependence in a tail (see, e.g., Joe (2014), Sect. 2.13).

One important problem in copula-based modeling is to decide which should be fitted to data of interest, a copula with symmetric tails or a copula with asymmetric tails. An additional problem arising from this is that if a copula with asymmetric tails is appropriate for the data, how much degree of tail asymmetry the copula should have. These problems are important because the lack of fit in tails of copulas leads to erroneous results in statistical analysis. For example, it is said that widespread applications of Gaussian copula, which has symmetric light tails, to financial products have contributed to the global financial crisis of 2008–2009 (see Donnelly and Embrechts (2010)). Therefore, in order to carry out decent statistical analysis, it is essential to evaluate the degree of tail asymmetry of copula appropriately. Given the stock market spooked by the outbreak of COVID-19, these problems would be even more important.

Some copula-based measures of tail asymmetry have been proposed in the literature. Nikoloulopoulos et al. (2012) and Dobrić et al. (2013) discussed a measure of tail asymmetry based on the difference between the conditional Spearman’s rhos for truncated data. Krupskii (2017) proposed an extension of their measure, which can regulate weights of tails. Rosco and Joe (2013) proposed three measures of tail asymmetry; two of them are based on moments or quantiles of a transformed univariate random variable and one of them is based on a difference between a copula and its reflected copula. As related works, measures of radial symmetry for the entire domain, not for tails, have been proposed, for example, by Dehgani et al. (2013) and Genest and Nešlehová (2014). See Joe (2014, Sect. 2.14) for the book treatment on this topic.

In this paper we propose a new copula-based measure of asymmetry between the lower and upper tails of bivariate distributions. The proposed measure and its sample analogues have various tractable properties; the proposed measure has a simple form and its calculation is fast; the proposed measure possesses desirable properties as a measure of tail asymmetry; the limits of the proposed measure as the index goes to the boundary of its domain can be easily evaluated under mild conditions on copulas; sample analogues of the proposed measure converge weakly to a Gaussian process or its mixture; simple methods for interval estimation and hypothesis testing based on the sample analogues are available; a multivariate extension of the proposed measure is straightforward.

The paper is organized as follows. In Sect. 2 we propose a new copula-based measure of tail asymmetry and present its basic properties. Section 3 considers the limits of our measure as the index goes to the boundary of its domain. Values of the proposed measure for some well-known copulas are discussed in Sect. 4. In Sect. 5 two sample analogues of the proposed measure are presented and their asymptotic properties are investigated. Also pointwise confidence intervals and simultaneous confidence regions of the proposed measure are discussed. In Sect. 6 a simulation study is carried out to consider the performance of the proposed sample analogues and confidence intervals. In Sect. 7 the proposed measure is compared with existing copula-based measures of tail asymmetry. In Sect. 8 the proposed measure is applied to daily returns of S&P500 and Nikkei225. A trivariate extension of the proposed measure and its sample analogue are considered in Sect. 9. Finally, concluding remarks and possible future work are discussed in Sect. 10.

Throughout this paper, a ‘copula’ refers to the bivariate case of a copula, namely, a bivariate cumulative distribution function with uniform [0, 1] margins, unless stated otherwise. Let $\mathscr {C}$ be a set of all the bivariate copulas. Let $\overline{C}$ denote the survival copula associated with *C*, which is defined by $\overline{C}(u_1,u_2) = 1 - u_1 - u_2 + C(u_1,u_2)$. Define $\bar{u}$ by $\bar{u}=1-u$.

## Definition and basic properties

In this section we propose a measure for comparing the probabilities of the lower and upper tails of bivariate distributions. The proposed measure is defined as follows.

### Definition 1

Let $(X_1,X_2)$ be an $\mathbb {R}^2$-valued random vector. Assume $X_1$ and $X_2$ have continuous margins $F_1$ and $F_2$, respectively. Then a measure of comparison between the lower-left and upper-right tail probabilities of $(X_1,X_2)$ is defined by

$$\begin{aligned} \alpha (u) = \log \left( \frac{ \mathbb {P}(F_1(X_1)> 1-u , F_2(X_2) > 1- u)}{\mathbb {P}(F_1(X_1) \le u , F_2(X_2) \le u)} \right) , \quad 0 < u \le 0.5. \end{aligned}$$

Here the definition of the logarithm function is extended to be $\log (x/y) = -\infty$ if $x=0$ and $y>0$, $\log (x/y) = \infty$ if $x>0$ and $y=0$, and $\log (x/y)=0$ if $x=y=0$.

Similarly it is possible to define a measure to compare the lower-right and upper-left tail probabilities of bivariate distributions. Properties of this measure immediately follow from those of $\alpha (u)$, which will be given hereafter, by replacing $(X_1,X_2)$ by $(X_1,-X_2)$.

The calculation of $\alpha (u)$ can be simplified if the distribution of $(X_1,X_2)$ is represented in terms of copula. The proof is straightforward and therefore omitted.

### Proposition 1

Let *C* denote a copula of $(X_1,X_2)$ given by $C(u_1,u_2) = \mathbb {P}(F_1$ $(X_1) \le u_1, F_2(X_2) \le u_2)$. Then $\alpha (u)$ defined in Definition 1 can be expressed as

1 $$\begin{aligned} \alpha (u) = \log \left( \frac{2u -1 + C(1-u,1-u)}{C(u,u)} \right) . \end{aligned}$$

Note that, using the survival copula $\overline{C}$ associated with *C* with $\bar{u} =1-u$, the proposed measure (1) has the simpler expression

$$\begin{aligned} \alpha (u) = \log \left( \frac{\overline{C}(\bar{u},\bar{u})}{C(u,u)} \right) . \end{aligned}$$

Throughout this paper, the lower $[0,u]^2$ tail and the upper $[1-u,1]^2$ tail of the copula *C* are said to be *symmetric* if $C(u,u) = \overline{C}(\bar{u},\bar{u})$.

Unlike many existing measures, the proposed measure (1) is not a global measure but a local one in the sense that this measure focuses on the probability of a subdomain of the copula regulated by the index *u*. Setting a particular value of *u* or looking at the behavior of $\alpha (u)$ for multiple choices of *u*, the proposed measure (1) provides a different insight from the global measure. For more details on the comparison between the proposed measure and existing ones, see Sect. 7.

It is straightforward to see that the following basic properties hold for $\alpha (u)$.

### Proposition 2

Let $\mathscr {C}$ be a set of all bivariate copulas. Denote the measure $\alpha (u)$ for the copula $C \in \mathscr {C}$ by $\alpha _C (u)$. Assume that $p_L=C(u,u)$, $p_U=\overline{C} (\bar{u},\bar{u})$, and $C_P(u,v) = C(v,u)$ is the permuted copula of *C*. Then, for $0<u \le 0.5$, we have that:

(i) $- \infty \le \alpha _C (u) \le \infty$ for every $C \in \mathscr {C}$; the equality holds only when either $p_U=0$ or $p_L=0$;
(ii) $\alpha _C (u) = 0$ if and only if $p_L=p_U$;
(iii) for fixed $p_U$, $\alpha _C(u)$ is monotonically non-increasing with respect to $p_L$; similarly, for fixed $p_L$, $\alpha _C(u)$ is monotonically non-decreasing with respect to $p_U$;
(iv) $\alpha _C (u) = - \alpha _{\overline{C}} (u)$ for every $C \in \mathscr {C}$;
(v) $$\alpha _{C_P}(u) = \alpha _{C}(u)$$ for every $$C \in \mathscr {C}$$;
(vi) if $C \in \mathscr {C}$ and $\{C_n\}_{n \in \mathbb {N}}$ is a sequence of copulas such that $C_n \rightarrow C$ uniformly, then $\alpha _{C_n} \rightarrow \alpha _C$.

Property (i) implies that the proposed measure is potentially unbounded although it is bounded except for the unusual case $p_U=0$ or $p_L=0$. Compared with a similar measure based on the difference between $p_U$ and $p_L$, our measure is advantageous in the sensitivity of detecting the asymmetry of tail probabilities for small *u* because the difference between $p_U$ and $p_L$ becomes small for $u \simeq 0$. Property (ii) implies that $\alpha _C(u) =0$ for any $0 < u \le 0.5$ if the copula *C* is radially symmetric, namely, $C \equiv \overline{C}$. Property (ii) is the same as an axiom of Dehgani et al. (2013) and is an extended property of Rosco and Joe (2013). Properties (iv)–(vi) are the same as the axioms of tail asymmetry presented in Sect. 2 of Rosco and Joe (2013). It is possible to use any function of $p_U/p_L$ other than the logarithm function as a measure of tail asymmetry. However one nice property of the proposed measure is property (iv) which other functions of $p_U/p_L$ do not have in general.

## Limits of the proposed measure

We consider limits of the proposed measure (1) as the index goes to the boundary of its domain. It follows from the expression (1) that $\alpha (0.5) = 0$ for any copula $C \in \mathscr {C}$. Therefore we have

2 $$\begin{aligned} \lim _{u \uparrow 0.5} \alpha (u) = 0. \end{aligned}$$

The limiting behavior of $\alpha (u)$ as $u \rightarrow 0$ is much more intricate. To consider this problem, define

3 $$\begin{aligned} \alpha (0) = \lim _{u \downarrow 0} \alpha (u), \end{aligned}$$

given that the limit exists. Here we present three expressions for the limit (3).

The first expression is based on the tail dependence coefficients. Tail dependence coefficients are often used as local dependence measures of bivariate distributions. The lower-left and upper-right tail dependence coefficients of the random variables $X_1$ and $X_2$ are defined by

$$\begin{aligned} \lambda _L = \lim _{u \downarrow 0} \frac{ \mathbb {P} (F_1(X_1) \le u, F_2(X_2) \le u)}{u} \quad \end{aligned}$$

and

$$\begin{aligned} \lambda _U = \lim _{u \uparrow 1} \frac{ \mathbb {P} (F_1(X_1)> u, F_2(X_2) > u)}{1-u}, \end{aligned}$$

respectively, given the limits exist. If $(X_1,X_2)$ has the copula *C*, the expressions for $\lambda _L$ and $\lambda _U$ are simplified as

4 $$\begin{aligned} \lambda _L = \lim _{u \downarrow 0} \frac{C(u,u)}{u} \quad \text{ and } \quad \lambda _U = \lim _{u \uparrow 1} \frac{\overline{C}(u,u)}{1-u}, \end{aligned}$$

respectively (see, e.g., Joe (2014), Sect. 2.13).

### Theorem 1

Let $(X_1,X_2)$ be an $\mathbb {R}^2$-valued random vector with the copula *C*. Assume that the lower-left and upper-right tail dependence coefficients of $X_1$ and $X_2$ exist and are given by $\lambda _L$ and $\lambda _U$, respectively. Suppose that either $\lambda _L$ or $\lambda _U$ is not equal to zero. Then

$$\begin{aligned} \alpha (0) = \log \left( \frac{\lambda _U}{\lambda _L} \right) . \end{aligned}$$

See Supplementary Material for the proof. Theorem 1 can be generalized by utilizing the concepts of tail orders and tail order parameters. If there exists a constant $\kappa _L > 0$ and a slowly varying function $\ell _L (u)$ such that $C(u,u) \sim u^{\kappa _L} \ell _L (u)$ $(u \rightarrow 0)$, then $\kappa _L$ is called the lower tail order of *C* and $\Upsilon _L=\lim _{u \downarrow 0} \ell _L (u)$ is called the lower tail order parameter of *C*, where $f(u) \sim g(u)$ $(u \rightarrow 0)$ is defined by $\lim _{u \downarrow 0} f(u)/g(u)=1$. Similarly, the upper tail order and the upper tail order parameter of *C* are defined by the lower tail order and the lower tail order parameter of the survival copula $\overline{C}$, respectively. See Joe (2014, Sect. 2.16) for more details on the tail orders and tail order parameters. Using the tail orders and tail order parameters, we have the following result. The proof is given in Supplementary Material.

### Theorem 2

Let $\kappa _L$ and $\kappa _U$ be the lower and upper tail orders of the copula *C*, respectively. Then $\alpha (0) = \infty$ if $\kappa _L < \kappa _U$ and $\alpha (0) = -\infty$ if $\kappa _L > \kappa _U$. If $\kappa _L=\kappa _U$ and either of the lower tail order parameter $\Upsilon _L$ or the upper tail order parameter $\Upsilon _U$ of *C* is not equal to zero, then $\alpha (0) = \log (\Upsilon _U / \Upsilon _L )$.

Note that Theorem 2 with $\kappa _L=\kappa _U=1$ reduces to Theorem 1. Theorems 1 and 2 are useful to evaluate $\alpha (0)$ if we already know the tail dependence coefficients, or tail orders and tail order parameters, of a copula. If those values are not known, the following third expression for $\alpha (0)$ could be useful.

### Theorem 3

Let $(X_1,X_2)$ be an $\mathbb {R}^2$-valued random vector with the copula *C*. Suppose that there exists $\varepsilon >0$ such that $\gamma (u) = d^2 C(t,t)/ dt^2 |_{t=u}$ exists in $(0,\varepsilon ) \cup (1-\varepsilon ,1)$. Assume that $\lim _{u \downarrow 0} d C(u,u) /du = \lim _{u \downarrow 0} d \overline{C}(\bar{u},\bar{u}) / du = 0$. Then

$$\begin{aligned} \alpha (0) = \log \left( \lim _{u \downarrow 0} \frac{\gamma (1-u)}{\gamma (u)} \right) , \end{aligned}$$

given the limit exists.

See Supplementary Material for the proof. As will be seen in the next section, Theorem 3 can be utilized to calculate $\alpha (0)$ for Clayton copula and Ali–Mikhail–Haq copula.

## Values of the proposed measure for some existing copulas

In this section we discuss the values of the proposed measure $\alpha (u)$ for some existing copulas. It is seen to be useful to plot $\alpha (u)$ with respect to *u* for comparing the probabilities of the lower $[0,u]^2$ tail and upper $[1-u,1]^2$ one for the whole range of $u \in (0,0.5]$. See, e.g., Joe (2014) for the definitions of the existing copulas discussed in this section.

### Copulas with symmetric tails

Proposition 2 implies that $\alpha (u)=0$ for any $u \in [0,0.5]$ if $C(u,u)=\overline{C}(\bar{u},\bar{u})$ for any $u \in [0,0.5]$. Such copulas include the independence copula, Gaussian copula, *t*-copula, Plackett copula and FGM copula. Among well-known Archimedean copulas, Frank copula has a radially symmetric shape and therefore $\alpha (u)=0$ for any *u*.

### Copulas with asymmetric tails

There exist various copulas for which $\alpha (u)$ is not equal to zero in general. Many Archimedean copulas have asymmetric tails, including Clayton copula, Gumbel copula, Ali–Mikhail–Haq copula and two-parameter BB copulas. In addition, some asymmetric extensions of Gaussian copula and *t*-copula have been proposed recently. Such extensions include the skew-normal copulas and skew-*t* copulas discussed in Joe (2006) and Yoshiba (2018), for which $\alpha (u)$ is not equal to zero in general. As examples of copulas with asymmetric tails, here we discuss the values of $\alpha (u)$ for the three well-known copulas, namely, Clayton copula, Ali–Mikhail–Haq copula and BB7 copula.

**Fig. 1:**
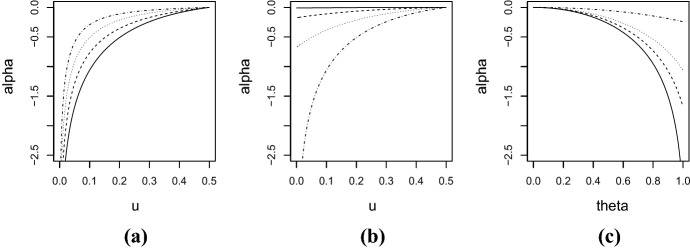
Plots of $\alpha (u)$ for: **a** Clayton copula (5) with respect to *u* for $\theta =1$ (solid), $\theta =5$ (dashed), $\theta =10$ (dotted), and $\theta =20$ (dotdashed), **b** Ali–Mikhail–Haq copula (6) with respect to *u* for $\theta =0.1$ (solid), $\theta =0.4$ (dashed), $\theta =0.7$ (dotted), and $\theta =1$ (dotdashed), and **c** Ali–Mikhail–Haq copula (6) with respect to $\theta$ for $u=0.01$ (solid), $u=0.05$ (dashed), $u=0.1$ (dotted), and $u=0.3$ (dotdashed)

*Clayton copula:* Clayton copula is defined by

5 $$\begin{aligned} C_{cl} (u_1,u_2; \theta ) = \max \left\{ u_1^{-\theta } + u_2^{-\theta } - 1 ,0 \right\} ^{-1/\theta }, \end{aligned}$$

where $\theta \in [-1,\infty ) \setminus \{0\}$. Fig. 1a plots the values of $\alpha (u)$ as a function of *u* for four positive values of $\theta$. (For an intuitive understanding of the distributions of Clayton copula, see Fig. S1a and b of Supplementary Material which plot random variates from Clayton copula with the two values of the parameters used in Fig. 1.) As is clear from equation (2), $\alpha (0.5)=0$ for any $\theta$. The smaller the value of *u*, the smaller the value of $\alpha (u)$. The figure also suggests that, for a fixed value of *u*, as $\theta$ increases, the value of $\alpha (u)$ approaches zero. The upper tail dependence coefficient of Clayton copula is 0 and the lower tail dependence coefficient is $2^{-1/\theta }$ for $\theta >0$ and 0 for $\theta \le 0$. Therefore, for $\theta > 0$, Theorem 1 implies that $\alpha (0) = -\infty$, meaning that the lower tail dependence is considerably stronger than the upper one. If $\theta \in [-1,0)$, it follows from Theorem 3 that $\alpha (0) = \infty$.

*Ali–Mikhail–Haq copula:* Ali–Mikhail–Haq copula is of the form

6 $$\begin{aligned} C(u_1,u_2) = \frac{u_1 u_2}{1-\theta (1-u_1)(1-u_2)}, \end{aligned}$$

where $\theta \in [-1,1]$. The values of $\alpha (u)$ as a function of *u* and $\theta$ are exhibited in Fig. 1b and c, respectively. (See Fig. S1c and d of Supplementary Material for plots of random variates generated from Ali–Mikhail–Haq copula with the two values of the parameters used in Fig. 1b.) Figure 1b suggests that $\alpha (u)$ decreases with *u*. Also it appears that, for a fixed value of *u*, the greater the value of $\theta$, the smaller the value of $\alpha (u)$. This observation can be seen more clearly in Fig. 1c which plots the values of $\alpha (u)$ as a function of $\theta$. Since both the lower and upper tail dependence coefficients of this copula are equal to zero, one can not apply Theorem 1 for the calculation of $\alpha (0)$. However Theorems 2 and 3 are applicable in this case and we have a simple form $\alpha (0) = \log (1-\theta ^2)$.

*BB7 copula:* Finally, consider the BB7 copula of Joe and Hu (1996) defined by

7 $$\begin{aligned} C(u_1,u_2) = 1 - \left[ 1- \left\{ \left( 1-\overline{u}_1^\theta \right) ^{-\delta } + \left( 1-\overline{u}_2^\theta \right) ^{-\delta } -1 \right\} ^{-1/\delta } \right] ^{1/\theta }, \end{aligned}$$

where $\delta >0$ and $\theta \ge 1$. Unlike the last two copulas, this model has two parameters. The parameter $\delta$ controls the lower tail dependence coefficient, while $\theta$ regulates the upper one. Indeed, the lower and upper tail dependence coefficients are known to be $2^{-1/\delta }$ and $2-2^{1/\theta }$, respectively.

**Fig. 2:**
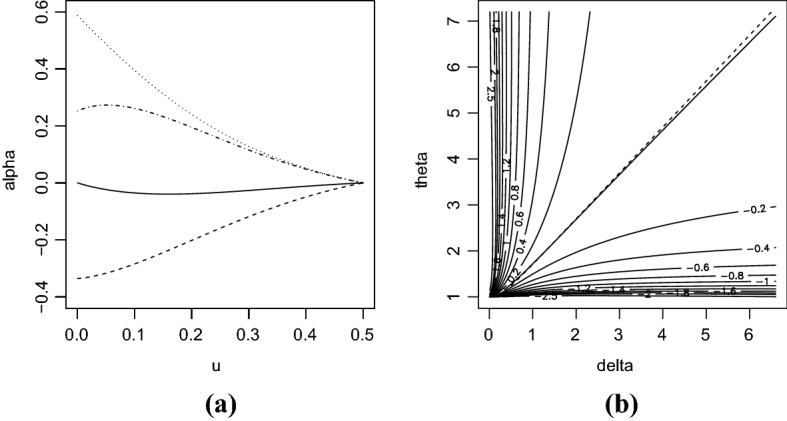
**a** Plot of $\alpha (u)$ for BB7 copula (7) with respect to *u* for $(\delta ,\theta )=(1,1.71)$ (solid), $(\delta ,\theta )=(1.94,1.71)$ (dashed), $(\delta ,\theta )=(1,7.27)$ (dotted), and $(\delta ,\theta )=(1.94,7.27)$ (dotdashed). **b** Contour plot of $\alpha (0.01)$ (solid) and plot of $\alpha (0)=0$ (dashed) with respect to $(\delta ,\theta )$

It follows from Theorem 1 that $\alpha (0)=\log (2-2^{1/\theta } ) - \delta ^{-1} \log 2$. Figure 2 displays a plot of $\alpha (u)$ with respect to *u* for four selected values of $(\delta ,\theta )$ and that of $\alpha (u)$ with respect to $(\delta ,\theta )$ for $u=0.01$. Note that, in Fig. 2a, $\delta =1$ and $\delta =1.94$ imply that the lower tail dependence coefficients are around 0.5 and 0.9, respectively, while $\theta =1.71$ and $\theta =7.27$ suggest that the upper tail dependence coefficients are about 0.5 and 0.7, respectively. (See also Fig. S1e–g of Supplementary Material for plots of random variates from BB7 copula (7) with the three combinations of the parameters in Fig. 2a.) Figure 2a suggests that, when both the lower and upper tail dependence coefficients are around 0.5, the values of $\alpha (u)$ are close to zero for any *u*. When the difference between the lower and upper tail dependence coefficients is large, $\alpha (u)$ appears to be monotonic with respect to *u*. It can be seen from Fig. 2b that the values of $\alpha (0.01)$ monotonically decreases as $\delta$ increases. Also, $\alpha (0.01)$ monotonically increases with $\theta$. The two contours $\alpha (0.01)=0$ and $\alpha (0)=0$ show somewhat similar shapes, implying that $\alpha (0.01)=0$ is a reasonable approximation to $\alpha (0)=0$.

## Two sample analogues of $\alpha (u)$

In practice, it is often the case that the form of the copula $C(u_1,u_2)$ underlying data is not known. In such a case, we need to estimate $\alpha (u)$ based on the data. In Sects. 5.1 and 5.2, we discuss a sample analogue of $\alpha (u)$ based on a sample from the copula. Section 5.3 presents a sample analogue of $\alpha (u)$ based on a sample from a distribution on $\mathbb {R}^2$.

### A sample analogue of $\alpha (u)$ based on a sample from a copula

A sample analogue of $\alpha (u)$ based on a sample from a copula is defined as follows.

#### Definition 2

Let $(U_{11},U_{21}), \ldots , (U_{1n},U_{2n})$ be a random sample from a copula. Then we define a sample analogue of $\alpha (u)$ by

$$\begin{aligned} \hat{\alpha }(u) = \log \left( \frac{T_U(u)}{T_L(u)} \right) , \end{aligned}$$

where

$$\begin{aligned} T_L(u)= & {} \frac{1}{n} \sum _{i=1}^n \varvec{1} (U_{1i} \le u, U_{2i} \le u),\\ T_U(u)= & {} \frac{1}{n} \sum _{i=1}^n \varvec{1} (U_{1i} \ge 1-u, U_{2i} \ge 1-u ), \end{aligned}$$

and $\varvec{1}(\cdot )$ is an indicator function, i.e., $\varvec{1}(A)=1$ if *A* is true and $\varvec{1}(A)=0$ otherwise.

In Sects. 5.1 and 5.2, we assume that $(U_{11},U_{21}),$ $\ldots ,$ $(U_{1n},U_{2n})$ is an iid sample from the copula $C(u_1,u_2)$. For iid $\mathbb {R}^2$-valued random vectors $(X_{11},X_{21})$ $,\ldots ,$ $(X_{1n},X_{2n})$, if the margins of $X_{11}$ and $X_{21}$ are known to be $F_1$ and $F_2$, respectively, then $\hat{\alpha }(u)$ can be obtained by replacing $(U_{1j},U_{2j})$ by $(F_1(X_{1j}),F_2(X_{2j}))$ $(j=1,\ldots ,n)$.

The goal of this subsection is to investigate some properties of $\hat{\alpha }(u)$. To achieve this, we first show the following lemma. See Supplementary Material for the proof.

#### Lemma 1

For $0 < u,v \le 0.5,$ we have the following:

$$\begin{aligned}&\mathbb {E} \left[ T_L(u) \right] = C_u, \quad \mathbb {E} \left[ T_U(u) \right] = \overline{C}_{\bar{u}}, \quad \mathrm{var} \left[ T_L (u) \right] = \frac{1}{n} C_u ( 1 - C_u ),\\&\mathrm{var} \left[ T_U (u) \right] = \frac{1}{n} \overline{C}_{\bar{u}} ( 1 - \overline{C}_{\bar{u}} ), \quad \mathrm{cov} \left[ T_L(u), T_L(v) \right] = \frac{1}{n} C_{u\wedge v} ( 1 - C_{u \vee v} ),\\&\mathrm{cov} \left[ T_L(u), T_U(v) \right] = -\frac{1}{n} C_{u} \overline{C}_{\bar{v}},\\&\mathrm{cov} \left[ T_U(u), T_U(v) \right] = \frac{1}{n} \overline{C}_{\bar{u} \vee \bar{v}} ( 1- \overline{C}_{\bar{u} \wedge \bar{v}} ), \end{aligned}$$

where $$u \wedge v = \min (u,v)$$, $$u \vee v = \max (u,v)$$, and $$C_w = C(w,w)$$.

This lemma implies that $\hat{\alpha }(u)$ is a consistent estimator of $\alpha (u)$. Applying this lemma, we obtain the following asymptotic result. The proof is given in Supplementary Material.

#### Theorem 4

Define

$$\begin{aligned} \mathbb {A}_n(u) = \sqrt{n} \left\{ \hat{\alpha }(u) - \alpha (u) \right\} , \quad 0 < u \le 0.5. \end{aligned}$$

Then, as $n \rightarrow \infty$, $\{ \mathbb {A}_n(u) \, | \, 0 < u \le 0.5 \}$ converges weakly to a centered Gaussian process with covariance function

8 $$\begin{aligned} \sigma (u,v) \equiv \mathbb {E} [ \mathbb {A}_n(u) \mathbb {A}_n(v) ] = \frac{C(u \vee v, u \vee v )+ \overline{C}(\bar{u} \wedge \bar{v}, \bar{u} \wedge \bar{v})}{C(u \vee v,u \vee v) \cdot \overline{C}(\bar{u} \wedge \bar{v}, \bar{u} \wedge \bar{v})}. \end{aligned}$$

We note that the covariance function (8) is monotonically decreasing with respect to $u \vee v$. If $u \vee v =0.5$, then the covariance function (8) reaches the minimum value 2/*C*(0.5, 0.5).

### Interval and region estimation based on $\hat{\alpha }(u)$

An asymptotic interval estimator of $\alpha (u)$ can be obtained by applying the asymptotic results obtained in the previous subsection. Theorem 4 implies that, for fixed $u \in (0, 0.5]$,

$$\begin{aligned} \sqrt{n} \left\{ \hat{\alpha }(u) - \alpha (u) \right\} \xrightarrow {d} N(0,\sigma ^2(u) ) \quad (n \rightarrow \infty ), \end{aligned}$$

where $\sigma ^2 (u)=\sigma (u,u)$ and $\sigma (u,u)$ is defined as in equation (8). Since $\sigma (u)$ includes the copula *C* which is usually not known in practice, we use an estimator of $\sigma (u)$ defined by

$$\begin{aligned} \hat{\sigma } (u) = \sqrt{ \frac{T_L(u) + T_U(u)}{T_L(u) \cdot T_U(u)}}. \end{aligned}$$

It follows from Lemma 1 that $\hat{\sigma } (u) \xrightarrow {a.s.} \sigma (u)$ as $n \rightarrow \infty$. Then we have $\sqrt{n} \{ \hat{\alpha }(u) - \alpha (u) \} / \hat{\sigma } (u) \xrightarrow {d} N(0,1)$ as $n \rightarrow \infty$. Hence a $100(1-p)$% nonparametric asymptotic confidence interval for $\alpha (u)$ is

9 $$\begin{aligned} \hat{\alpha }(u) - \frac{ z_{p/2} \hat{\sigma } (u) }{\sqrt{n}} \le \alpha (u) \le \hat{\alpha }(u) + \frac{ z_{p/2} \hat{\sigma } (u) }{\sqrt{n}}, \end{aligned}$$

where $z_{p/2}$ satisfies $\mathbb {P}(Z \ge z_{p/2}) =p/2$, $Z \sim N(0,1)$, and $0< p < 1$.

For multiple values of the index, say $u_1,\ldots ,u_m$, some methods are available to construct simultaneous confidence regions of $(\alpha (u_1),\ldots ,\alpha (u_m))$. Bonferroni correction is a well-known method for this purpose. However the use of Bonferroni correction based on (9) leads to the confidence region that is too wide. In addition, when Bonferroni correction is adopted, the dependence among $\{ \hat{\alpha }(u_1),\ldots ,\hat{\alpha }(u_m) \}$ is not taken into account for the resulting simultaneous regions. Given the weak convergence to the Gaussian process as shown in Theorem 4, a possible solution is to construct asymptotic simultaneous confidence regions based on the asymptotic Gaussianity of $(\hat{\alpha }(u_1),\ldots ,\hat{\alpha }(u_m))$. To achieve this, we first show the the following result. See Supplementary Material for the proof.

#### Corollary 1

Let $\varvec{a}= \sqrt{n} \{ \hat{\alpha }(u_1) - \alpha (u_1), \ldots , \hat{\alpha }(u_m) - \alpha (u_m) \}^T$ and $u_1< \cdots < u_m$. Suppose

$$\begin{aligned} \hat{\varvec{\Sigma }} = \left( \begin{array}{cccc} \hat{\sigma }^2(u_1) &{} \hat{\sigma } (u_1,u_2) &{} \ldots &{} \hat{\sigma } (u_1,u_m) \\ \hat{\sigma } (u_1,u_2) &{} \hat{\sigma }^2 (u_2) &{} \ldots &{} \hat{\sigma } (u_2,u_m) \\ \vdots &{} \vdots &{} \ddots &{} \vdots \\ \hat{\sigma } (u_1,u_m) &{} \hat{\sigma } (u_2,u_m) &{} \ldots &{} \hat{\sigma }^2 (u_m) \end{array} \right) , \end{aligned}$$

where $\hat{\sigma }^2 (u_i) = \hat{\sigma } (u_i,u_i)$ and $\hat{\sigma } (u_i,u_j) = \{T_L(u_j) + T_U(u_j) \}/\{T_L(u_j) T_U(u_j)\}$ $(i \le j)$. Assume that $\hat{\varvec{\Sigma }}$ is invertible. Then

$$\begin{aligned} \varvec{a}^T \hat{\varvec{\Sigma }}^{-1} \varvec{a} \xrightarrow {d} \chi ^2_m \quad \text{ as } n \rightarrow \infty , \end{aligned}$$

where $\chi ^2_m$ denotes the chi-squared distribution with *m* degrees of freedom.

Using this result, an asymptotic $100(1-p)$% simultaneous confidence region for $(\alpha (u_1),\ldots ,\alpha (u_m))$ is given by

10 $$\begin{aligned} \left\{ (\alpha (u_1),\ldots ,\alpha (u_m)) \, ; \, \varvec{a}^T \hat{\varvec{\Sigma }}^{-1} \varvec{a} \le \chi ^2_{m,1-p} \right\} , \end{aligned}$$

where $\chi ^2_{m,1-p}$ denotes the $100(1-p)$th percentile of the chi-squared distribution with *m* degrees of freedom. Unlike the simultaneous confidence region based on Bonferroni correction which has a rectangular shape, the confidence region (10) is ellipsoidal in shape.

If *n* is small or $u_j \simeq 0$ for some *j*, the asymptotic simultaneous confidence region (10) could be considerably different from the true simultaneous confidence region because the asymptotic theory may not be applicable. In that case, the $100(1-p)$th percentile $\chi ^2_{m,1-p}$ in (10) could be replaced with the percentile estimated from bootstrap samples under the assumption of elliptical symmetry of $(\hat{\alpha }(u_1),\ldots ,\hat{\alpha }(u_m))$ (see Davison and Hinkley (1997), Sect. 5.8). Alternatively, one can adopt other general bootstrap methods such as the one proposed by Mandel and Betensky (2008) if the dependence among $\{ \hat{\alpha }(u_1),\ldots ,\hat{\alpha }(u_m) \}$ is not strong.

We note that some hypothesis tests can be established using the results of this subsection. For example, for a given function $\alpha _0(u)$, it is possible to consider the test $H_0: \alpha (u) = \alpha _0 (u)$ against $H_1: \alpha (u) \ne \alpha _0(u)$ for $u = u_1,\ldots ,u_m$. This can be done, for example, by using $\varvec{a}^T \hat{\varvec{\Sigma }}^{-1} \varvec{a}$ in Corollary 1 as the test statistic and substituting $\alpha _0(u)$ into $\alpha (u)$ in $\varvec{a}^T \hat{\varvec{\Sigma }}^{-1} \varvec{a}$. In particular, if $\alpha _0(u)=0$, one can establish a test of tail symmetry.

### A sample analogue of $\alpha (u)$ based on a sample from a distribution on $\mathbb {R}^2$

The sample analogue of $\alpha (u)$ given in Definition 2 can be calculated on the assumption that the margins of the $\mathbb {R}^2$-valued random vector are known. Here we discuss the case in which margins are unknown and empirical distributions are adopted as the margins.

#### Definition 3

Let $(X_{11},X_{21}), \ldots , (X_{1n},X_{2n})$ be $\mathbb {R}^2$-valued random vectors. Then we define a sample analogue of $\alpha (u)$ by

$$\begin{aligned} \hat{\alpha }^* (u) = \log \left( \frac{T^*_U(u)}{T^*_L(u)} \right) , \end{aligned}$$

where

11 $$\begin{aligned} T^*_L(u)= & {} \frac{1}{n} \sum _{i=1}^n \varvec{1} ( \hat{F}_1(X_{1i}) \le u, \hat{F}_2 (X_{2i}) \le u ),\nonumber \\ T^*_U(u)= & {} \frac{1}{n} \sum _{i=1}^n \varvec{1} ( \hat{F}_1(X_{1i}) \ge 1-u, \hat{F}_2(X_{2i}) \ge 1-u ),\nonumber \\ \hat{F}_j (X_{ji})= & {} \frac{1}{n+1} \sum _{k=1}^n \varvec{1} ( X_{jk} \le X_{ji} ), \quad j=1,2. \end{aligned}$$

Note that the denominator of the empirical distribution function (11) is defined by $n+1$ rather than *n* in order to avoid positive bias of $\hat{\alpha }(u)$.

The following result implies that the value of $\hat{\alpha }^*(u)$ is small for $u = 0.5$. The proof is given in Supplementary Material.

#### Theorem 5

If *n* is even, then $\mathbb {P} (\hat{\alpha }^* (0.5)=0)=1$. If *n* is odd, it holds that

12 $$\begin{aligned} \mathbb {P} \left( \log \left( 1-\frac{1}{n T_L^*(0.5)} \right) \le \hat{\alpha }^* (0.5) \le \log \left( 1+\frac{1}{n T_L^*(0.5)} \right) \right) =1. \end{aligned}$$

The authors have not yet obtained the asymptotic distribution for $\hat{\alpha }^*(u)$. However the following results are available regarding $T_U^*(u)$ and $T_L^*(u)$. The proof is straightforward from Fermanian et al. (2004), Tsukahara (2005) and Segers (2012) and therefore omitted.

#### Proposition 3

Let $(X_{11},X_{21}),\ldots ,(X_{1n},X_{2n})$ be iid random vectors with the copula *C*(*u*, *v*) and the continuous margins. Assume that *C*(*u*, *v*) is differentiable with continuous *i*-th partial derivatives $(i=1,2)$. Then, as $n \rightarrow \infty$,

$$\begin{aligned}&\sqrt{n} \left\{ T^*_L(u) - C(u,u) \right\} \xrightarrow {d} D^C (u),\\&\sqrt{n} \left\{ T^*_U(u) - \overline{C}(\bar{u},\bar{u}) \right\} \xrightarrow {d} D^{\overline{C}}(\bar{u}), \end{aligned}$$

where

$$\begin{aligned} D^C(u) = U(u,u) - \frac{ \partial C(u_1,u)}{\partial u_1} \biggr |_{u_1=u} U(u,1) - \frac{ \partial C(u,u_2)}{\partial u_2} \biggr |_{u_2=u} U(1,u), \end{aligned}$$

*U* is a centered Gaussian process with covariance function

$$\begin{aligned} \mathbb {E} [U(u_1,u_2) U(v_1,v_2) ] = C( u_1 \wedge v_1, u_2 \wedge v_2 ) - C(u_1,u_2) C(v_1,v_2), \end{aligned}$$

and $u \wedge v$ is defined as in Lemma 1.

Confidence intervals for $\alpha (u)$ can be numerically constructed using the bootstrap method. Theorem 5 implies that the confidence intervals are narrow when *u* is close to 0.5. Hypothesis tests can also be established based on the bootstrap confidence intervals. It should be noted that, in order to calculate $\alpha (u)$ based on bootstrap samples, $\hat{F}_1$ and $\hat{F}_2$ in (11) should be calculated based on each bootstrap sample. If $\hat{F}_1$ and $\hat{F}_2$ are calculated from the original data, the bootstrap confidence intervals become similar to the asymptotic confidence intervals (9) for large *n*. Simultaneous confidence regions for $(\alpha (u_1), \ldots , \alpha (u_m))$ and related hypothesis tests are also available by using the bootstrap methods discussed in the last two paragraphs of the former subsection.

## Simulation study

We carry out a simulation study to compare the performance of the two proposed sample analogues of $\alpha (u)$. Also, another experiment is given to discuss the range of the index *u* in which $\hat{\alpha }(u)$ and its asymptotic confidence interval (9) are reasonably applicable.

First, in order to compare the performance of the two proposed sample analogues of $\alpha (u)$, we consider the following cumulative distribution function

13 $$\begin{aligned} F(x_1,x_2) = C_{cl}(F_1(x_1),F_2(x_2); 20), \quad -\infty< x_1,x_2 < \infty , \end{aligned}$$

where $F_j(x)$ is the cumulative distribution function of the standard Cauchy distribution, i.e., $F_j(x) = 0.5+ \pi ^{-1} \arctan x$, and $C_{cl}(u_1,u_2;\theta )$ denotes the Clayton copula (5).

**Fig. 3:**
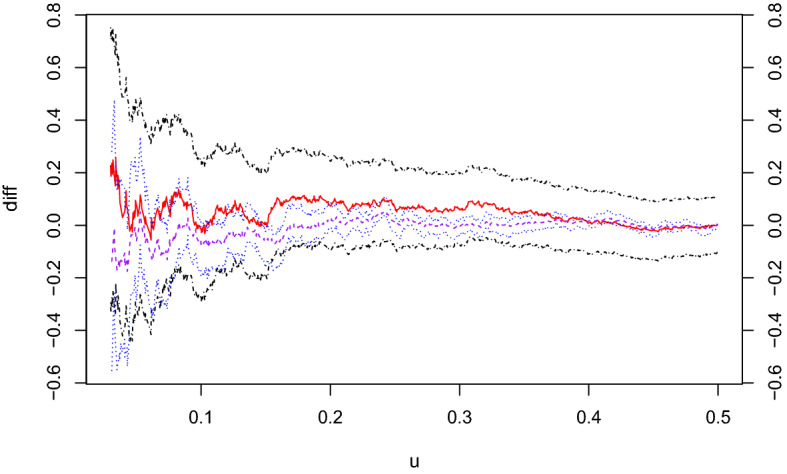
Plot of $\hat{\alpha }(u)-\alpha (u)$ (solid, red), $\hat{\alpha }^*(u) - \alpha (u)$ (dashed, purple), the lower and upper bounds of asymptotic 90$\%$ confidence intervals of $\hat{\alpha }(u) - \alpha (u)$ (dotdashed, black), and the lower and upper bounds of bootstrap 90% confidence intervals of $\hat{\alpha }^*(u)-\alpha (u)$ (dotted, blue) obtained from a sample of size 1000 from the distribution (13). (Color figure online)

**Fig. 4:**
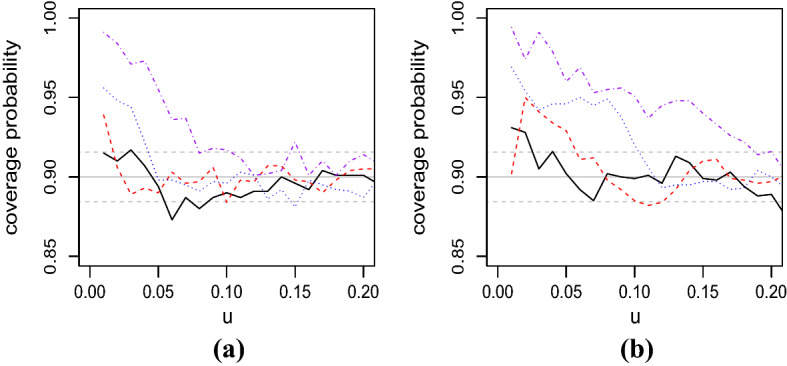
Coverage probabilities of the asymptotic 90% confidence intervals (9) of $\alpha (u)$ for $u=0.01j$ $(j=1,\ldots ,20)$, calculated from 1000 samples of size $n=100$ (dotdashed, purple), $n=250$ (dotted, blue), $n=1000$ (dashed, red), and $n=5000$ (solid, black) from Clayton copula (5) with: **a** $\theta =20$ and **b** $\theta =1$. The solid and dashed horizontal lines in gray represent the nominal coverage probability and the bounds of its 90% confidence intervals, respectively. (Color figure online)

Figure 3 plots the values of $\hat{\alpha }(u)-\alpha (u)$, $\hat{\alpha }^*(u) - \alpha (u)$ and their bounds of 90% confidence intervals for a sample of size $n=1000$ from the distribution (13). See also Fig. 1a for the plot of $\alpha (u)$. For the calculations of $\hat{\alpha }(u)$ and its confidence intervals (9), the sample $\{(x_{1i},x_{2i})\}$ is transformed into the copula sample $\{(u_{1i},u_{2i})\}$ via $u_{ji} = F_j(x_{ji})$, where $F_j$ is the true margin $(j=1,2)$. The confidence intervals based on $\hat{\alpha }^*(u)$ are calculated using the basic bootstrap method based on 999 resamples of size 1000; see the last paragraph of Sect. 5.3 for details. The minimum value of *u* in the plot is set to be $u_\mathrm{min} = 0.03$; see Fig. 4a and related discussion below.

Figure 3 suggests that, when *u* is around 0.15 or greater, both $\hat{\alpha }(u)$ and $\hat{\alpha }^*(u)$ are stably not far from the true value. If *u* is less than 0.15, the differences between the sample analogues and the true value become variable. Actually, for $u \le 0.15$, the smaller the value of *u*, the wider the ranges of the confidence intervals based on both $\hat{\alpha }(u)$ and $\hat{\alpha }^*(u)$. The confidence intervals based on $\hat{\alpha }^*(u)$ are generally narrower than those based on $\hat{\alpha }(u)$. This tendency is particularly obvious for $u \simeq 0.5$, where the bounds of the confidence intervals based on $\hat{\alpha }^*(u)$ are close to zero; see Theorem 5.

Our additional experiments suggest that the confidence intervals based on $\hat{\alpha }^*(u)$ are much narrower than those based on $\hat{\alpha }(u)$ for $u \simeq 0.5$ for other simulated datasets as well. When *u* is not close to 0.5, it is not necessarily the case that the confidence intervals based on $\hat{\alpha }^*(u)$ are narrower than those based on $\hat{\alpha }(u)$; see Sect. 8.

Next we consider another simulation study to find the range of the index *u* in which $\hat{\alpha }(u)$ and its asymptotic confidence interval (9) are reasonably applicable. Figure 4 displays the coverage probabilities of the asymptotic 90% confidence intervals of $\alpha (u)$, calculated from 1000 samples of size *n* from Clayton copula $C_{cl}(u_1,u_2;\theta )$ for some selected values of the parameter $\theta$ and sample size *n*. In the figure, the nominal coverage probability is set to be 0.9 and the bounds of its 90% confidence intervals are calculated as $0.9 \pm z_{0.1/2} \{ 0.1 \times (1-0.1) \times 1000 \}^{1/2}$ using the normal approximation of the binomial distribution. (See Fig. S1a and b of Supplementary Material for plots of random variates from the two Clayton copulas used in Fig. 4.)

Figure 4 implies that, when $u \simeq 0.2$, the coverage probabilities are generally within their 90% confidence intervals. When *u* is not close to 0.2, the coverage probabilities are significantly different from 0.9 for some combinations of $(n,\theta )$. A general trend is that, as *u* decreases, the number of cases in which the coverage probabilities are significantly greater than 0.9 increases. In addition, for fixed *u* and $\theta$, the coverage probability is more likely to be within its 90% confidence interval for a greater value of *n*. In particular, for $n=5000$, the performance of the asymptotic confidence intervals is generally satisfactory for any $u \, (\ge 0.01)$. On the other hand, if *n* is equal to 100, the asymptotic confidence intervals tend to show poor performance except for the case $\theta =20$ and $u \le 0.07$. Comparing Fig. 4a and b, the performance of the asymptotic confidence intervals for $\theta =20$ seems better than that for $\theta =1$. It appears from Fig. 4 and the discussion above that the selection of the minimum value of *u*, in which the asymptotic theory is applicable, depends on *n* and the underlying distribution.

A possible conjecture induced from the figure is that there exists a certain relationship between the numbers of observations in both tails and a practical range of *u*. This can be investigated by calculating the values of $n \cdot \min \{ C_{cl}(u,u;\theta ),$ $\overline{C}_{cl}(\bar{u},\bar{u};\theta ) \} (\equiv T_{\min }(u,n,\theta ))$ for the combinations of $(u,n,\theta )$ for which the asymptotic confidence intervals show satisfactory performance. The values of $\min _{u} T_{\min }(u,n,\theta )$ for each $(n,\theta )$ range from 5.3 and 8.7, and it seems that asymptotic theory is available for fairly small values of $\min _{u} T_{\min }(u,n,\theta )$ for the two Clayton copulas.

## Comparison with existing measures

In this section we compare our measure with existing copula-based measures of tail asymmetry. Rosco and Joe (2013) proposed three measures of tail asymmetry. One of their measures based on the distance between a copula *C* and its survival copula is defined by

14 $$\begin{aligned} \varsigma _3 = \sup _{(u_1,u_2) \in [0,1]^2 } \left\{ \left| C(u_1,u_2) - \overline{C}(\bar{u}_1,\bar{u}_2) \right| \right\} . \end{aligned}$$

This measure has also been proposed by Dehgani et al. (2013) as a limiting case of a measure of radial asymmetry for bivariate random variables.

Our measure (1) has some similarities to and differences from the measure (14). Similarities include that both are functions of a copula *C* and its survival function. Also, both measures satisfy Properties (ii), (v) and (vi) of Proposition 2.

However there are considerable differences between the two measures (1) and (14). First, the domains of a copula the two measures evaluate are different. The measure (14) is a global measure in the sense that the whole domain of the copula is taken into account to evaluate the value of the measure, while our measure (1) is a local measure which focuses on squared subdomains of the copula. By choosing the value of the index *u*, our measure (1) enables us to choose the subdomain of a copula which analysts are interested in. However the prescription for selecting the value of *u* is not always straightforward and the choice of the index *u* could influence the results of analysis. The index-free measure (14) does not have such a problem. However the supremum value of this measure is not necessarily attained in the tails of the distribution and the value of the measure might not reflect the tail probabilities if $u_1 \ge 0.5$ or $u_2 \ge 0.5$. Also, because of its locality, computations associated with our measure (1) are very fast.

In addition there are differences between the two measures (1) and (14) in terms of properties. Our measure (1) satisfies all the properties of (i)–(vi) of Proposition 2 which include four (out of five) axioms of Rosco and Joe (2013). However this measure does not satisfy one of the axioms, i.e., axiom (i), of Rosco and Joe (2013) and therefore the value of the measure could be unbounded for special cases. The measure (14) also satisfies four axioms of Rosco and Joe (2013) including the axiom (i). On the other hand, the measure (14) does not satisfy their axiom (iii) which is equivalent to Property (iv) of Proposition 2, implying that the measure (14) does not distinguish which tail probability is greater than the other one.

The other two measures of Rosco and Joe (2013) are derived through different approaches. For a bivariate random vector $(U_1,U_2)$ from a copula, the two measures are based on the moments or quantile function of the univariate random variable $U_1+U_2-1$. Therefore these measures are essentially different from ours which is based on the joint distribution of the bivariate random vector $(U_1,U_2)$.

Another copula-based measure for tail asymmetry has been proposed by Krupskii (2017). It is defined by

15 $$\begin{aligned} \varrho _{K} (a,u) = \varrho _L (a,u) - \varrho _U (a,u), \end{aligned}$$

where $0<u \le 0.5$, *a* is a weighting function,

$$\begin{aligned} \varrho _L (a,u)= & {} \mathrm{cor} \left[ \left. a \left( 1 - \frac{U_1}{u} \right) , a \left( 1 - \frac{U_2}{u} \right) \right| U_1< u , U_2 < u \right] ,\\ \varrho _U (a,u)= & {} \mathrm{cor} \left[ \left. a \left( 1 - \frac{1-U_1}{u} \right) , a \left( 1 - \frac{1-U_2}{u} \right) \right| U_1> 1-u , U_2 > 1-u \right] . \end{aligned}$$

If $a(x)=x$, the measure (15) reduces to the measure discussed by Nikoloulopoulos et al. (2012) and Dobrić et al. (2013). Properties of each term of the measure (15) have been investigated by Krupskii and Joe (2015).

The measure (15) is related to ours in the sense that the values of their measures are calculated from the subdomain of a copula indexed by the truncation parameter. However the measure (15) is based on Spearman’s rhos or correlation coefficients of a truncated copula, and therefore the interpretation of the values of the measure (15) is essentially different from ours. A nice property of the measure (15) is that the weights of tails can be controlled through the weight function *a*. Therefore this measure can be a useful measure of tail asymmetry if the weight function is appropriately defined.

## Example

As an application of the proposed measure, we consider a dataset of daily returns of two stock indices. The dataset is taken from historical data in Yahoo Finance, available at https://finance.yahoo.com/quote/%5EGSPC/history/ and https://finance.yahoo.com/quote/%5EN225/history/. We consider stock daily returns of S&P500 and Nikkei225 observed from the 1st of April, 2008 until the 31st of March, 2013, inclusive. We fit the autoregressive-generalized autoregressive conditional heteroscedastic model AR(1)-GARCH(1,1) to each of the stock daily returns using ugarchfit in ‘rugarch’ package in R (R Core Team 2020; Ghalanos 2020). The Student *t*-distribution is used as the conditional density for the innovations. We consider the residuals $\{(x_{1i} , x_{2i}) \}_{i=1}^n$ $(n=1180)$ of the fitted AR(1)-GARCH(1,1), where $x_{1i}$ and $x_{2i}$ are the residuals of S&P500 and Nikkei225, respectively. The residuals show unexpected changes in daily return which are not explained by the model; if the joint plunging probability is higher than the joint soaring probability, then the proposed measures $\alpha (u)$ is supposed to be negative.

**Fig. 5:**
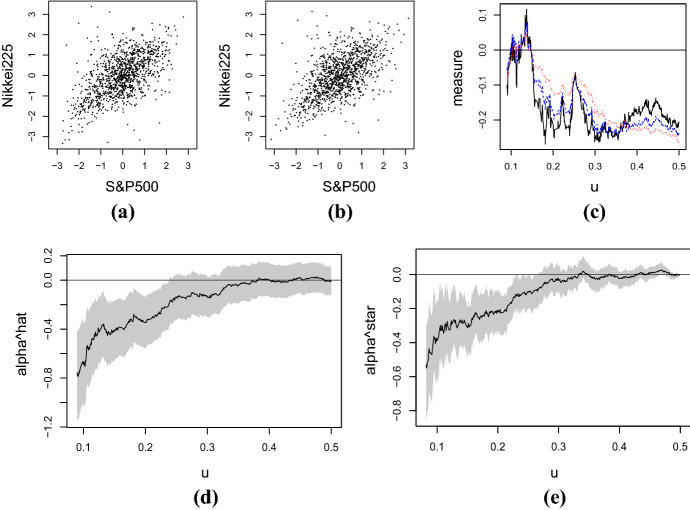
Plots of $\{(\mathrm{\Phi }^{-1}(u_{1i}),\mathrm{\Phi }^{-1}(u_{2i}))\}_{i=1}^{1180}$, where $\{ (u_{1i},u_{2i}) \}$ is the copula sample transformed from the residuals via the cumulative distribution functions of: **a** Student *t*-distribution and **b** empirical distribution. **c** Plot of $- \varrho _K(a,u)$, a modified version of the measure (15) of Krupskii (2017), with: $a(x)=x$ (solid, black), $a(x)=x^2$ (dashed, blue), and $a(x)=x^4$ (dotted, red). Plots of the proposed measure (black) and its asymptotic or bootstrap 90% confidence intervals (gray) for: **d** $\hat{\alpha }(u)$ and **e** $\hat{\alpha }^* (u)$. (Color figure online)

We discuss $\hat{\alpha }(u)$ defined in Definition 2 and $\hat{\alpha }^*(u)$ defined in Definition 3. In order to obtain the copula sample $\{ (u_{1i},u_{2i}) \}$ for $\hat{\alpha }(u)$, we transform the residuals $\{(x_{1i},x_{2i})\}$ via $(u_{1i},u_{2i}) = (F_1 (x_{1i}) , F_2(x_{2i}))$, where $F_1$ and $F_2$ are the cumulative distribution functions of Student *t*-distribution estimated using the maximum likelihood method. We assume, though not mathematically precise, that $F_1$ and $F_2$ are known. Figure 5a plots $\{ ({\Phi }^{-1} (u_{1i}), {\Phi }^{-1} (u_{2i})) \}$ in which the residuals are transformed into $\{ (u_{1i},u_{2i}) \}$ via the cumulative distribution functions of Student *t*-distribution. Here ${\Phi }^{-1}$ denotes the inverse of the cumulative distribution function of the standard normal. The values of $\hat{\alpha }(u)$ calculated from the sample and their 90% asymptotic confidence intervals (9) are displayed in Fig. 5d. In this frame, the minimum value of *u* is set to be $\min \{ u \in (0,0.5] ; T_L(u) ,T_U(u) \ge 30/n \} \simeq 0.090$ in order that the asymptotic theory is applicable.

For the calculation of $\hat{\alpha }^*(u)$, we use the empirical distribution functions (11) to transform the residuals $\{(x_{1i},x_{2i})\}$ into the copula sample $\{ (u_{1i},u_{2i}) \}$. The transformed sample is displayed in Fig. 5b. The values of $\hat{\alpha }^*(u)$ calculated from the sample are plotted in Fig. 5e. The same frame also plots the 90% confidence intervals based on 999 resamples of size 1180 using the basic bootstrap method. The minimum value of *u* in the plot is $u^*_\mathrm{min}= \min \{ u \in (0,0.5] ; T_L^*(u) ,T_U^*(u) \ge 30/n \} \simeq 0.082$.

Figure 5a and b suggest that there are more observations in the lower-left tail than the upper-right one. In order to investigate whether this tail asymmetry is significant or not, we consider Fig. 5d and e showing the values of $\hat{\alpha }(u)$ and $\hat{\alpha }^*(u)$, respectively. These frames imply that $\hat{\alpha }(u)$ and $\hat{\alpha }^*(u)$ are negative in most areas of the domain of *u*, suggesting that the lower tail probability is greater than the upper one for most values of $u \in (u_\mathrm{min},0.5]$. In particular the two frames imply the general tendency that, for $u \le 0.33$, $\hat{\alpha }(u)$ and $\hat{\alpha }^*(u)$ increase with *u*. Both asymptotic and bootstrap 90% confidence intervals do not contain nonnegative values for any $u \le 0.22$. This implies that the tails are asymmetric in the sense that the lower $[0,u]^2$ tail probability is greater than the upper $[1-u,1]^2$ one. On the other hand, when *u* is greater than 0.27, both 90% confidence intervals include 0. It is not clear to judge the existence of asymmetry in the tail probabilities for $u \in (0.22,0.27]$. For a pre-specified value, say $u_0$, of *u*, the discussion above can be applied to hypothesis testing by adopting the 90% confidence intervals as the acceptance regions of the level 0.9 test of $H_0: \alpha (u_0)=0$ against $H_1: \alpha (u_0) \ne 0$.

As seen in the discussion above, both $\hat{\alpha }(u)$ and $\hat{\alpha }^*(u)$ show similar tendencies in general. Actually, the two data plots given in Fig. 5a and b look similar at the first glance. However Fig. 5d and e reveal that there are some differences between $\hat{\alpha }(u)$ and $\hat{\alpha }^*(u)$. For example, the values of $\hat{\alpha }(u)$ are generally smaller than those of $\hat{\alpha }^*(u)$ at least for $u \in (0,0.15]$. Also the bootstrap confidence intervals based on $\hat{\alpha }^*(u)$ are narrower than the asymptotic confidence intervals based on $\hat{\alpha }(u)$ for large *u* as implied in Theorem 5.

Apart from the tests based on pointwise confidence intervals given in Fig. 5d and e, we carry out a different test for a nominal size of 0.1 based on the test statistic in Corollary 1. We test $H_0: \alpha (u) = 0$ against $H_1: \alpha (u) \ne 0$ for $\{ u; u= u_\mathrm{min} + (0.15-u_\mathrm{min}) j / 4, \ j=0,\ldots ,4\}$. The test statistic is $T=\varvec{a}^T \hat{\varvec{\Sigma }}^{-1} \varvec{a} \simeq 13.52$ with $\mathbb {P} (T > 13.52) \simeq 0.019 < 0.1$. Therefore we reject the null hypothesis that the lower $[0,u]^2$ tail and upper $[1-u,1]^2$ tail are symmetric for the 5 equally spaced points of *u* in $[u_\mathrm{min},0.15]$.

We apply other measures of tail asymmetry to the copula sample displayed in Fig. 5a. The measure (14) of Rosco and Joe (2013) is calculated as $\hat{\varsigma }_3 \simeq 0.043$. Another measure we consider here is a modified version of Krupskii ’s (2017) measure (15), namely, $- \varrho _K(a,u)$. This modification is made to interpret the sign of the measure in the same manner as in that of ours. Figure 5c displays the estimates of $-\varrho _K(a,u)$ with respect to *u* for the three specific functions of *a*. The three curves of the modified measure $-\varrho _K(a,u)$ show somewhat similar trends. For example, the three curves agree that there is stronger correlation in the lower $[0,u]^2$ tail than the upper $[1-u,1]^2$ one for $u \ge 0.15$. This is somewhat similar to the result based on our measure as well.

We summarize the results of the analysis of stock daily return data. The results based on the proposed measures suggest that the lower $[0,u]^2$ tail probability is greater than the upper $[1-u,1]^2$ one for most values of $u \in (u_{\mathrm{min}},0.5]$, where $u_{\mathrm{min}} \simeq 0.053$. In particular, there is significant difference between the lower and upper tail probabilities for $u \le 0.22$. From the economic perspective, this result implies that the joint plunging probability is higher than the joint soaring probability with the threshold $u \le 0.22$. Therefore it is recommended to use a copula with asymmetric tails for an appropriate modeling of the residuals of the daily return data appropriately. The three cases of the measure of Krupskii (2017) agree that, for $u \ge 0.15$, there is stronger correlation in the lower $[0,u]^2$ tail than in the upper $[1-u,1]^2$ one.

In order to see whether this tendency for the stock daily returns in 2008–2013 holds in another period, a similar analysis can be conducted to the daily returns of S&P500 and Nikkei225 observed from the 1st of April, 2014 until the 31st of March, 2019, inclusive. Our analysis suggests that $\hat{\alpha }(u)$ is negative for $u \le 0.17$ and all the three cases of the measure (15) of Krupskii (2017) are negative for $u \le 0.10$. However the plot of $\hat{\alpha }^*(u)$ as well as the 90% asymptotic confidence intervals based on $\hat{\alpha }(u)$ do not suggest clear tendency of tail asymmetry.

## A trivariate extension of $\alpha (u)$ and its sample analogue

The proposed measure $\alpha (u)$ for bivariate random vectors can be extended to a measure for trivariate random vectors as follows.

### Definition 4

Let $(X_1,X_2,X_3)$ be an $\mathbb {R}^3$-valued random vector. Suppose $X_j$ has the continuous margin $F_j$ $(j=1,2,3)$. Then we define a measure of comparison between the lower and upper tail probability of $(X_1,X_2,X_3)$ by

$$\begin{aligned} {\varvec{\alpha }_3 (u)} =&\log \left( \frac{ \mathbb {P}(F_1(X_1)> 1-u , F_2(X_2)> 1- u , F_3(X_3) > 1-u)}{\mathbb {P}(F_1(X_1) \le u , F_2(X_2) \le u , F_3(X_3) \le u)} \right) , \\&\qquad \qquad \qquad \qquad \qquad \qquad \qquad \qquad \qquad \qquad \qquad 0 < u \le 0.5, \end{aligned}$$

where the logarithm function is defined as in Definition 1.

As the following proposition shows, the trivariate extension ${\varvec{\alpha }}_3 (u)$ can be represented in terms of copulas. The proof is straightforward and omitted.

### Proposition 4

Define a copula of $(X_1,X_2,X_3)$ by $C_3$, namely, $C_3(u_1,u_2,u_3) = \mathbb {P}(F_1(X_1) \le u_1, F_2(X_2) \le u_2, F_3 (X_3) \le u_3)$. Then ${\varvec{\alpha }}_3 (u)$ defined in Definition 4 has the expression

$$\begin{aligned} {\varvec{\alpha }}_3 (u) = \log \left( \frac{ 1 - 3 \bar{u} + C_3(\bar{u},\bar{u},1) + C_3(\bar{u},1,\bar{u}) + C_3(1,\bar{u},\bar{u}) - C_3(\bar{u},\bar{u},\bar{u}) }{C_3(u,u,u)} \right) . \end{aligned}$$

It can also be seen that the measure ${\varvec{\alpha }}_3(u)$ has properties similar to those in Proposition 2; see Proposition S1 of Supplementary Material for details. A difference between the bivariate measure $\alpha (u)$ in Definition 1 and the trivariate extension ${\varvec{\alpha }}_3 (u)$ in Definition 4 is the value of the measure at $u=0.5$. Unlike the bivariate measure, the value ${\varvec{\alpha }}_3(0.5)$ is not equal to 0 in general.

Extending the results in Sect. 5.1, here we briefly discuss a sample analogue of ${\varvec{\alpha }}_3 (u)$ based on a sample from a copula.

### Definition 5

Let $(U_{11},U_{21},U_{31}), \ldots , (U_{1n},U_{2n},U_{3n})$ be a random sample from a trivariate copula. Then a sample analogue of ${\varvec{\alpha }}_3 (u)$ is defined by

$$\begin{aligned} \hat{\varvec{\alpha }}_3 (u) = \log \left( \frac{T_{3U}(u)}{T_{3L}(u)} \right) , \end{aligned}$$

where

$$\begin{aligned} T_{3L}(u)= & {} \frac{1}{n} \sum _{i=1}^n \varvec{1} (U_{1i} \le u, U_{2i} \le u, U_{3i} \le u),\\ T_{3U}(u)= & {} \frac{1}{n} \sum _{i=1}^n \varvec{1} (U_{1i} \ge 1-u, U_{2i} \ge 1-u , U_{3i} \ge 1-u ), \end{aligned}$$

and $\varvec{1}(\cdot )$ is as in Definition 2.

In a similar manner as in Lemma 1, it can be proved that $\hat{\varvec{\alpha }}_3 (u)$ is a consistent estimator of $\varvec{\alpha }_3 (u)$. Also the weak convergence to a Gaussian process also holds for $\hat{\varvec{\alpha }}_3 (u)$; see Theorem S1 of Supplementary Material. This result can be utilized to establish inferential methods based on $\hat{\varvec{\alpha }}_3 (\varvec{u})$ by following the discussion in Sect. 5.2.

## Discussion

In this paper we have proposed a copula-based measure of asymmetry between the lower and upper tail probabilities. It has been seen that the proposed measure has some properties which are desirable as a measure of tail asymmetry. Sample analogues of the proposed measure have been presented, and statistical inference based on them, including point, interval and region estimation and related hypothesis testing, has been shown to be very simple. The practical importance of the proposed measure has been demonstrated through statistical analysis of stock return data.

This paper discusses measures for bivariate and trivariate data. It is straightforward to extend the proposed measures to a *d*-variate one by generalizing the idea in Definition 4 as follows. Let $(X_1, \ldots , X_d)$ be an $\mathbb {R}^d$-valued random vector with continuous univariate margins. Then a *d*-dimensional extension of the proposed measure for $(X_1, \ldots , X_d)$ is

$$\begin{aligned} {\varvec{\alpha }_d (u)}&= \log \left( \frac{ \mathbb {P}(F_1(X_1)> 1-u , \ldots , F_d(X_d) > 1-u)}{\mathbb {P}(F_1(X_1) \le u , \ldots , F_d(X_d) \le u)} \right) , \quad 0 < u \le 0.5, \end{aligned}$$

where $F_j$ denotes the cumulative distribution function of $X_j$ $(j=1,\ldots ,d)$. Some properties of this measure can be obtained by following the results in Sect. 9.

Another multivariate extension of the proposed bivariate measure is available by adopting the approach in Embrechts et al. (2016) and Hofert and Koike (2019). With this approach, an extended measure of tail asymmetry for $(X_1, \ldots , X_d)$ is defined by

$$\begin{aligned} {\varvec{A}}_d(\varvec{U}) = \left( \begin{array}{cccc} \alpha _{11} (u_{11}) &{} \alpha _{12}(u_{12}) &{} \cdots &{} \alpha _{1d} (u_{1d}) \\ \alpha _{21} (u_{21}) &{} \alpha _{22}(u_{22}) &{} \cdots &{} \alpha _{2d} (u_{2d}) \\ \vdots &{} \vdots &{} \ddots &{} \vdots \\ \alpha _{d1}(u_{d1}) &{} \alpha _{d2}(u_{d2}) &{} \ldots &{} \alpha _{dd}(u_{dd}) \end{array} \right) , \end{aligned}$$

where ${\varvec{U}}=(u_{ij})$, $\alpha _{ij}$ is the proposed measure (1) of the random vector $(X_i,X_j) \ (i,j=1,\ldots ,d)$. This general dimensional measure essentially consists of the proposed bivariate measures (1) applied to every pair of variables. The properties of each element of the measure ${\varvec{A}}_d({\varvec{U}})$ are straightforward from the results of this paper. The measure ${\varvec{A}}_d({\varvec{U}})$ might be used to discuss joint tail behaviour when there are more than two variables. It would be a possible topic for future work to investigate properties of this extended measure as a matrix and evaluate the values of the measure for multivariate copulas such as some examples of the vine copulas (Aas et al. 2009; Czado 2010).

## Electronic supplementary material

The online version of this article contains the following supplementary materials, which are available to authorized users:

Supplementary Material: Supplementary Material (362_2022_1297_MOESM3_ES M.pdf) contains the proofs of Lemma 1, Theorems 1–5 and Corollary 1; plots of random variates from the copulas discussed in Sects. 4.2 and 6; and some theoretical results related to the measure for trivariate random vectors and its sample analogue proposed in Section 9.
R codes: The R code (362_2022_1297_MOESM2_ESM.r) is for reproducing the simulation study in Sect. 6 and the applied example in Sect. 8. The other R code (362_2022_1297_MOESM1_ESM.r) defines functions used in 362_2022_1297_MOESM2_ESM.r.

## Supplementary Information

Below is the link to the electronic supplementary material.

Supplementary file 1 (r 11 KB)

Supplementary file 2 (r 13 KB)

Supplementary file 3 (pdf 2533 KB)
