# Supplementary material for: Copula-based measures of asymmetry between the lower and upper tail probabilities
Source: Stat Pap (Berl). 2022 Mar 6;63(6):1907–29. doi: 10.1007/s00362-022-01297-w (PMC8898571; doi:10.1007/s00362-022-01297-w)
Supplement: Supplementary file 3 — (pdf 2533 KB) [file 362_2022_1297_MOESM3_ESM.pdf]

Noname manuscript No.
(will be inserted by the editor)

Supplementary Material for "Copula-based measures of asymmetry between the lower and upper tail probabilities"

Shogo Kato · Toshinao Yoshiba · Shinto Eguchi

Received: 1 February, 2022

The Supplementary Material is organized as follows. Section S1 presents the proofs of Lemma 1, Theorems 1–5 and Corollary 1 of the article. Section S2 displays plots of random variates from Clayton copula, Ali-Mikhail-Haq copula and BB7 copula discussed in Sections 4.2 and 6 of the article. Finally, Section S3 presents some properties of the measure for trivariate random vectors and its sample analogue discussed in Section 9 of the article.

S1 Proofs

S1.1 Proof of Theorem 1

Proof It follows from the expression (4) that

$$\lambda_U = \lim_{u \uparrow 1} \frac{\overline{C}(u,u)}{1-u} = \lim_{u \downarrow 0} \frac{\overline{C}(\bar{u},\bar{u})}{u}.$$

This result and Proposition 4 imply that

$$\alpha(0) = \lim_{u \downarrow 0} \log \left(\frac{\overline{C}(\bar{u},\bar{u})}{C(u,u)} \right) = \log \left(\lim_{u \downarrow 0} \frac{\overline{C}(\bar{u},\bar{u})/u}{C(u,u)/u} \right) = \log \left(\frac{\lambda_U}{\lambda_L} \right).$$

Kato's research was supported by JSPS KAKENHI Grant Numbers JP17K05379 and JP20K03759.

Shogo Kato
Institute of Statistical Mathematics, 10-3 Midori-cho, Tachikawa, Tokyo 190-8562, Japan
Tel.: +81-80-5533-8500
E-mail: skato@ism.ac.jp

Toshinao Yoshiba
Tokyo Metropolitan University, Graduate School of Management, 18F 1-4-1 Marunouchi, Chiyoda-ku, Tokyo, 100-0005, Japan

Shinto Eguchi
Institute of Statistical Mathematics, 10-3 Midori-cho, Tachikawa, Tokyo 190-8562, Japan

The last equality holds because λ_U and λ_L exist and either of λ_U and λ_L is not equal to zero.

S1.2 Proof of Theorem 2

Proof If follows from the assumption that there exists a slowly varying function $\ell_L(u)$ such that $C(u,u) \sim u^{\kappa_L}\ell_L(u)$ as $u \to 0$. Similarly, there exists a slowly varying function $\ell_U(u)$ such that $\overline{C}(\bar{u},\bar{u}) \sim u^{\kappa_U}\ell_U(u)$ as $u \to 0$. Therefore

$$\alpha(0) = \lim_{u\downarrow 0} \log\left(\frac{\overline{C}(\bar{u},\bar{u})}{C(u,u)}\right) = \lim_{u\downarrow 0} \log\left(u^{\kappa_U-\kappa_L}\frac{\ell_U(u)}{\ell_L(u)}\right)$$
$$= \begin{cases} \infty, & \kappa_U > \kappa_L, \\ -\infty, & \kappa_U < \kappa_L. \end{cases}$$

The last equality holds because $\ell_U(u)/\ell_L(u)$ is slowly varying. If $\kappa_U = \kappa_L$ and either $\varUpsilon_U \neq 0$ or $\varUpsilon_L \neq 0$, then

$$\alpha(0) = \lim_{u\downarrow 0} \log\left(\frac{\ell_U(u)}{\ell_L(u)}\right) = \log\left(\lim_{u\downarrow 0}\frac{\ell_U(u)}{\ell_L(u)}\right) = \log\left(\frac{\varUpsilon_U}{\varUpsilon_L}\right).$$

S1.3 Proof of Theorem 3

Proof Proposition 4 implies that $\alpha(0)$ can be expressed as

$$\alpha(0) = \lim_{u\downarrow 0} \log\left(\frac{\overline{C}(\bar{u},\bar{u})}{C(u,u)}\right) = \log\left(\lim_{u\downarrow 0}\frac{\overline{C}(\bar{u},\bar{u})}{C(u,u)}\right).$$

Since $\lim_{u\downarrow 0} dC(u,u)/du = \lim_{u\downarrow 0} d\overline{C}(\bar{u},\bar{u})/du = 0$, the l'Hôpital's rule is applicable to the last expression of the equation above. Hence we have

$$\alpha(0) = \log\left(\lim_{u\downarrow 0}\frac{d^2\overline{C}(\bar{u},\bar{u})/du^2}{d^2C(u,u)/du^2}\right) = \log\left(\lim_{u\downarrow 0}\frac{\gamma(1-u)}{\gamma(u)}\right)$$

as required.

S1.4 Proof of Lemma 1

Proof It is straightforward to see that $\mathbb{E}\left[T_L(u)\right]$ and $\mathrm{var}\left[T_L(u)\right]$ can be calculated as

$$\mathbb{E}\left[T_L(u)\right] = \mathbb{E}\left[\frac{1}{n}\sum_{i=1}^{n}\mathbf{1}(U_{1i} \leq u, U_{2i} \leq u)\right] = \frac{1}{n}\sum_{i=1}^{n}\mathbb{E}\left[\mathbf{1}(U_{1i} \leq u, U_{2i} \leq u)\right]$$
$$= \frac{1}{n}\cdot nC_u = C_u,$$

$$\begin{aligned}
\operatorname{var}\left[T_L(u)\right] &= \operatorname{var}\left[\frac{1}{n}\sum_{i=1}^{n}\mathbf{1}(U_{1i}\le u, U_{2i}\le u)\right]\\
&= \frac{n}{n^2}\operatorname{var}\left[\mathbf{1}(U_{11}\le u, U_{21}\le u)\right]\\
&= \frac{1}{n}\left(\mathbb{E}\left[\mathbf{1}^2(U_{11}\le u, U_{21}\le u)\right] - \left\{\mathbb{E}\left[\mathbf{1}(U_{11}\le u, U_{21}\le u)\right]\right\}^2\right)\\
&= \frac{1}{n}\left(C_u - C_u^2\right) = \frac{1}{n}C_u(1-C_u).
\end{aligned}$$

Noting that $\mathbb{E}\left[\mathbf{1}(U_{11}>1-u, U_{21}>1-u)\right] = \overline{C}_{\bar{u}}$, the other expectation and variance, namely, $\mathbb{E}\left[T_U(u)\right]$ and $\operatorname{var}\left[T_U(u)\right]$, can be calculated in a similar manner.

Consider

$$\operatorname{cov}\left[T_L(u), T_L(v)\right] = \mathbb{E}\left[T_L(u)T_L(v)\right] - \mathbb{E}\left[T_L(u)\right]\mathbb{E}\left[T_L(v)\right].$$

The first term of the right-hand side of the equation above is

$$\begin{aligned}
\mathbb{E}\left[T_L(u)T_L(v)\right] &= \frac{1}{n^2}\mathbb{E}\left[\sum_{i=1}^{n}\mathbf{1}(U_{1i}\le u, U_{2i}\le u)\sum_{j=1}^{n}\mathbf{1}(U_{1j}\le v, U_{2j}\le v)\right]\\
&= \frac{1}{n^2}\sum_{i,j=1}^{n}\mathbb{E}\left[\mathbf{1}(U_{1i}\le u, U_{2i}\le u)\mathbf{1}(U_{1j}\le v, U_{2j}\le v)\right]\\
&= \frac{1}{n^2}\sum_{i=1}^{n}\mathbb{E}\left[\mathbf{1}(U_{1i}\le u, U_{2i}\le u)\mathbf{1}(U_{1i}\le v, U_{2i}\le v)\right]\\
&\quad + \frac{1}{n^2}\sum_{i\ne j}\mathbb{E}\left[\mathbf{1}(U_{1i}\le u, U_{2i}\le u)\mathbf{1}(U_{1j}\le v, U_{2j}\le v)\right]\\
&= \frac{1}{n^2}\sum_{i=1}^{n}\mathbb{E}\left[\mathbf{1}(U_{1i}\le u\wedge v, U_{2i}\le u\wedge v)\right]\\
&\quad + \frac{1}{n^2}\sum_{i\ne j}\mathbb{E}\left[\mathbf{1}(U_{1i}\le u, U_{2i}\le u)\right]\mathbb{E}\left[\mathbf{1}(U_{1j}\le v, U_{2j}\le v)\right]\\
&= \frac{1}{n^2}\left\{nC_{u\wedge v} + n(n-1)C_uC_v\right\} = \frac{1}{n}C_{u\wedge v}\left\{1+(n-1)C_{u\vee v}\right\}.
\end{aligned}$$

Therefore we have

$$\operatorname{cov}\left[T_L(u), T_L(v)\right] = \frac{1}{n}C_{u\wedge v}\left\{1+(n-1)C_{u\vee v}\right\} - C_uC_v = \frac{1}{n}C_{u\wedge v}(1-C_{u\vee v}).$$

Similarly, $\mathrm{cov}\,[T_U(u), T_U(v)]$ can be calculated. The other covariance $\mathrm{cov}[T_L(u), T_U(v)]$ can also be obtained via a similar approach, but notice that

$$\begin{aligned}\mathbb{E}\,[T_L(u)T_U(v)] &= \frac{1}{n^2}\sum_{i=1}^{n}\mathbb{E}\,[\mathbf{1}(U_{1i}\le u, U_{2i}\le u)\mathbf{1}(U_{1i}>1-v, U_{2i}>1-v)]\\ &\quad+\frac{1}{n^2}\sum_{i\neq j}\mathbb{E}\,[\mathbf{1}(U_{1i}\le u, U_{2i}\le u)\mathbf{1}(U_{1j}>1-v, U_{2j}>1-v)]\\ &= 0+\frac{n(n-1)}{n^2}C_u\overline{C}_{\bar{v}} = \frac{n-1}{n}C_u\overline{C}_{\bar{v}}.\end{aligned}$$

The second equality holds because $0 < u, v \le 0.5$. Thus

$$\mathrm{cov}\,[T_L(u), T_U(v)] = \mathbb{E}\,[T_L(u)T_U(v)] - \mathbb{E}\,[T_L(u)]\,\mathbb{E}\,[T_U(v)] = -\frac{1}{n}C_u\overline{C}_{\bar{v}}.$$

S1.5 Proof of Theorem 4

Proof Without loss of generality, assume $0 < u \le v \le 0.5$. Let

$$\hat{\boldsymbol{\beta}} = (\hat{\beta}_1, \hat{\beta}_2, \hat{\beta}_3, \hat{\beta}_4)^T = (T_L(u), T_U(u), T_L(v), T_U(v))^T,$$

$$\boldsymbol{\beta} = (\beta_1, \beta_2, \beta_3, \beta_4)^T = (C(u,u), \overline{C}(\bar{u},\bar{u}), C(v,v), \overline{C}(\bar{v},\bar{v}))^T,$$

$$\boldsymbol{\Sigma}_{\boldsymbol{\beta}} = (\sigma_{\beta ij})_{i,j}, \quad \sigma_{\beta ij} = n\cdot\mathrm{cov}(\hat{\beta}_i, \hat{\beta}_j).$$

Then it follows from Lemma 1 and the central limit theorem that

$$\sqrt{n}(\hat{\boldsymbol{\beta}} - \boldsymbol{\beta}) \xrightarrow{d} N(\mathbf{0}, \boldsymbol{\Sigma}_{\boldsymbol{\beta}}) \quad \text{as } n \to \infty.$$

Define

$$h(\boldsymbol{\beta}) = \begin{pmatrix}\log(\beta_2/\beta_1)\\ \log(\beta_4/\beta_3)\end{pmatrix} = \begin{pmatrix}\alpha(u)\\ \alpha(v)\end{pmatrix}.$$

Applying the delta method, we have

$$\sqrt{n}\left\{h(\boldsymbol{\beta}) - h(\hat{\boldsymbol{\beta}})\right\} \xrightarrow{d} N\left(0, \nabla h(\boldsymbol{\beta})^T\boldsymbol{\Sigma}_{\boldsymbol{\beta}}\nabla h(\boldsymbol{\beta})\right) \quad \text{as } n \to \infty,$$

where

$$\nabla h(\boldsymbol{\beta}) = \begin{pmatrix}\frac{\partial}{\partial\beta_1}\log(\beta_2/\beta_1) & \frac{\partial}{\partial\beta_1}\log(\beta_4/\beta_3)\\ \vdots & \vdots\\ \frac{\partial}{\partial\beta_4}\log(\beta_2/\beta_1) & \frac{\partial}{\partial\beta_4}\log(\beta_4/\beta_3)\end{pmatrix} = \begin{pmatrix}-1/\beta_1 & 0\\ 1/\beta_2 & 0\\ 0 & -1/\beta_3\\ 0 & 1/\beta_4\end{pmatrix}.$$

The asymptotic variance can be calculated as

$$
\begin{aligned}
&\nabla h(\boldsymbol{\beta})^T \boldsymbol{\Sigma}_{\boldsymbol{\beta}} \nabla h(\boldsymbol{\beta}) \\
&= \begin{pmatrix} \frac{\sigma_{11}}{\beta_1^2} - \frac{2\sigma_{12}}{\beta_1\beta_2} + \frac{\sigma_{22}}{\beta_2^2} & \frac{\sigma_{13}}{\beta_1\beta_3} - \frac{\sigma_{23}}{\beta_2\beta_3} - \frac{\sigma_{14}}{\beta_1\beta_4} + \frac{\sigma_{24}}{\beta_2\beta_4} \\ \frac{\sigma_{13}}{\beta_1\beta_3} - \frac{\sigma_{23}}{\beta_2\beta_3} - \frac{\sigma_{14}}{\beta_1\beta_4} + \frac{\sigma_{24}}{\beta_2\beta_4} & \frac{\sigma_{33}}{\beta_3^2} - \frac{2\sigma_{34}}{\beta_3\beta_4} + \frac{\sigma_{44}}{\beta_4^2} \end{pmatrix} \\
&= \begin{pmatrix} \frac{C(u,u)+\overline{C}(\bar{u},\bar{u})}{C(u,u)\cdot\overline{C}(\bar{u},\bar{u})} & \frac{C(v,v)+\overline{C}(\bar{v},\bar{v})}{C(v,v)\cdot\overline{C}(\bar{v},\bar{v})} \\ \frac{C(v,v)+\overline{C}(\bar{v},\bar{v})}{C(v,v)\cdot\overline{C}(\bar{v},\bar{v})} & \frac{C(v,v)+\overline{C}(\bar{v},\bar{v})}{C(v,v)\cdot\overline{C}(\bar{v},\bar{v})} \end{pmatrix} \\
&= \begin{pmatrix} \sigma(u,u) & \sigma(u,v) \\ \sigma(u,v) & \sigma(v,v) \end{pmatrix}. \qquad \text{(S1)}
\end{aligned}
$$

The case $0 < v < u \leq 0.5$ can be calculated in the same manner. Then, for any $0 < u, v \leq 0.5$, it follows that, as $n \to \infty$, $(\mathbb{A}_n(u), \mathbb{A}_n(v))(= \sqrt{n}\{h(\boldsymbol{\beta}) - h(\hat{\boldsymbol{\beta}})\})$ converges weakly to the two-dimensional Gaussian distribution with mean 0 and the covariance matrix (S1). Weak convergence of $(\mathbb{A}_n(u_1), \ldots, \mathbb{A}_n(u_m))$ to an m-dimensional centered Gaussian distribution for $u_1, \ldots, u_m \in (0, 0.5]$ $(u_i \neq u_j,\ i \neq j)$ can be shown in a similar manner. Therefore $\{\mathbb{A}_n(u) \mid 0 < u \leq 0.5\}$ converges weakly to a centered Gaussian process with covariance function $\sigma(u, v)$ as $n \to \infty$.

S1.6 Proof of Corollary 1

Proof Theorem 4 implies that $\boldsymbol{a}$ converges weakly to an m-dimensional normal distribution $N(\mathbf{0}, \boldsymbol{\Sigma})$ as n tends to infinity, where

$$
\boldsymbol{\Sigma} = \begin{pmatrix} \sigma^2(u_1) & \sigma(u_1, u_2) & \ldots & \sigma(u_1, u_m) \\ \sigma(u_1, u_2) & \sigma^2(u_2) & \ldots & \sigma(u_2, u_m) \\ \vdots & \vdots & \ddots & \vdots \\ \sigma(u_1, u_m) & \sigma(u_2, u_m) & \ldots & \sigma^2(u_m) \end{pmatrix},
$$

$\sigma^2(u_i) = \sigma(u_i, u_i)$, and $\sigma(u_i, u_j)$ is defined as in Theorem 4. Then we have $\boldsymbol{a}^T \boldsymbol{\Sigma}^{-1} \boldsymbol{a} \xrightarrow{d} \chi^2_m$ as $n \to \infty$. Since $T_L(u_j)$ and $T_U(u_j)$ are consistent estimators of $C(u_j, u_j)$ and $\overline{C}(\bar{u}_j, \bar{u}_j)$, respectively, it holds that, for any (i, j), $\hat{\sigma}(u_i, u_j)$ converges in probability to $\sigma(u_i, u_j)$ as $n \to \infty$. It then follows from Slutsky's theorem that $\boldsymbol{a}^T \hat{\boldsymbol{\Sigma}}^{-1} \boldsymbol{a} \xrightarrow{d} \chi^2_m$ as $n \to \infty$.

S1.7 Proof of Theorem 5

It can be seen that

$$
\begin{aligned}
T_L^*(0.5) &= \frac{1}{n} \sum_{i=1}^{n} \mathbf{1}(\hat{F}_1(X_{1i}) \leq 0.5) - \mathbf{1}(\hat{F}_1(X_{1i}) \leq 0.5, \hat{F}_2(X_{2i}) > 0.5) \\
&= \frac{1}{n} \left\lceil \frac{n}{2} \right\rceil - \frac{1}{n} \sum_{i=1}^{n} \mathbf{1}(\hat{F}_1(X_{1i}) \leq 0.5, \hat{F}_2(X_{2i}) > 0.5),
\end{aligned}
$$

where $\lceil x \rceil = \min\{y \in \mathbb{Z};\, y \geq x\}$. The second equality follows from the fact that the ordered values of the empirical distribution functions $\hat{F}_1(X_{11}), \ldots, \hat{F}_1(X_{1n})$ are equally spaced between $[1/(n+1), n/(n+1)]$. Similarly, we have

$$T_U^*(0.5) = \frac{1}{n}\left\lceil \frac{n}{2} \right\rceil - \frac{1}{n}\sum_{i=1}^{n} \mathbf{1}(\hat{F}_1(X_{1i}) < 0.5, \hat{F}_2(X_{2i}) \geq 0.5).$$

These results imply that

$$|T_U^*(0.5) - T_L^*(0.5)| = \frac{1}{n}\left| \sum_{i=1}^{n} \mathbf{1}(\hat{F}_1(X_{1i}) = 0.5, \hat{F}_2(X_{2i}) > 0.5) \right. \\ \left. - \mathbf{1}(\hat{F}_1(X_{1i}) < 0.5, \hat{F}_2(X_{2i}) = 0.5) \right|.$$

If n is even, $\hat{F}_1(X_{1i})$ and $\hat{F}_2(X_{2i})$ do not take values in 0.5 and hence $\mathbb{P}(T_U^*(0.5) - T_L^*(0.5) = 0) = \mathbb{P}(\hat{\alpha}^*(0.5) = 0) = 1$. If n is odd, the number of the random variables $\{\hat{F}_j(X_{ji})\}_{i=1}^n$ which take values in 0.5 is 0 or 1 for each $j = 1, 2$. This implies that

$$\mathbb{P}\left(|T_U^*(0.5) - T_L^*(0.5)| \leq \frac{1}{n} \right) = 1. \tag{S2}$$

Also,

$$\begin{aligned} &\mathbb{P}\left(|T_U^*(0.5) - T_L^*(0.5)| \leq \frac{1}{n} \right) \\ &= \mathbb{P}\left(T_L^*(0.5) - \frac{1}{n} \leq T_U^*(0.5) \leq T_L^*(0.5) + \frac{1}{n} \right) \\ &= \mathbb{P}\left(\log\left(\frac{T_L^*(0.5) - \frac{1}{n}}{T_L^*(0.5)} \right) \leq \log\left(\frac{T_U^*(0.5)}{T_L^*(0.5)} \right) \leq \log\left(\frac{T_L^*(0.5) + \frac{1}{n}}{T_L^*(0.5)} \right) \right) \\ &= \mathbb{P}\left(\log\left(1 - \frac{1}{nT_L^*(0.5)} \right) \leq \hat{\alpha}^*(0.5) \leq \log\left(1 + \frac{1}{nT_L^*(0.5)} \right) \right). \end{aligned} \tag{S3}$$

It follows from (S2) and (S3) that (12) holds as required.

S2 Plots of random variates from some existing copulas

Fig. S1 plots random variates from Clayton copula (5), Ali-Mikhail-Haq copula (6) and BB7 copula (7) with some selected values of the parameter(s). This figure is given to help an intuitive understanding of the distributions of those copulas discussed in the paper.

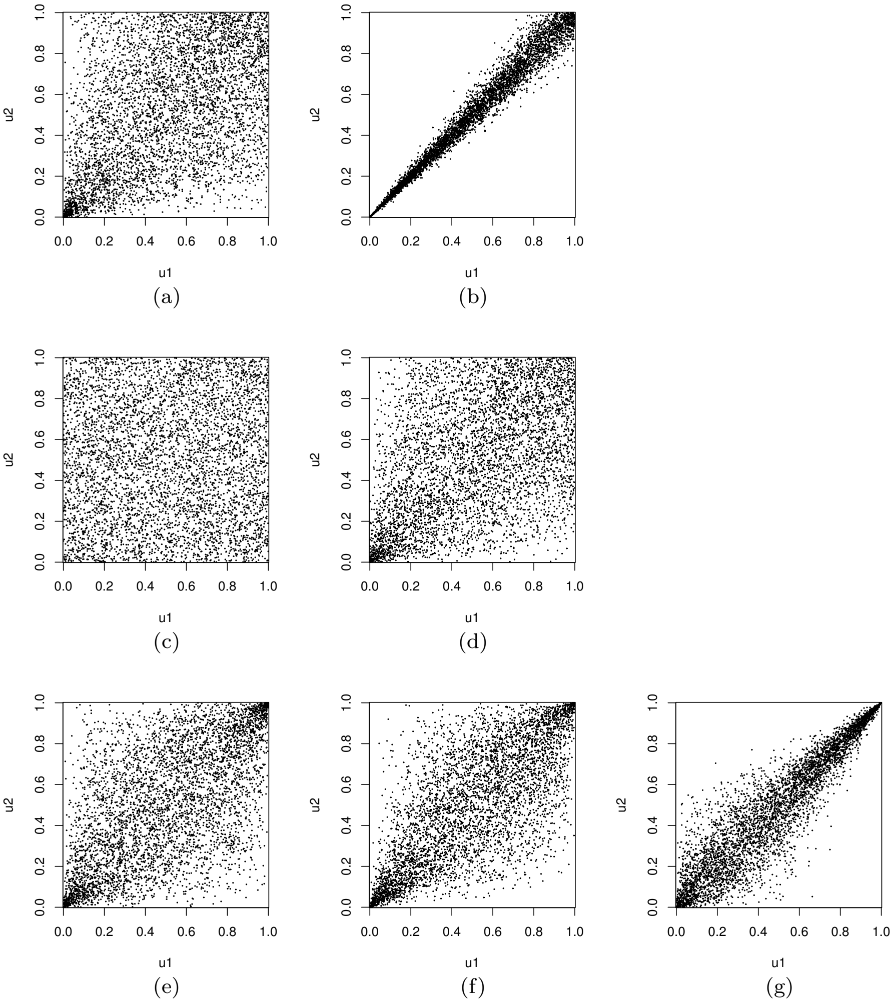

Fig. S1 Plots of 5000 random variates from: Clayton copula (5) with (a) $\theta = 1$ and (b) $\theta = 20$; Ali-Mikhail-Haq copula (6) with (c) $\theta = 0.1$ and (e) $\theta = 1$; and BB7 copula (7) with (f) $(\delta, \theta) = (1, 1.71)$, (g) $(\delta, \theta) = (1.94, 1.71)$ and (h) $(\delta, \theta) = (1, 7.27)$.

S3 Some properties of the trivariate measure $\boldsymbol{\alpha}_3(u)$ and its sample analogue

Here we present some properties of the trivariate extension $\boldsymbol{\alpha}_3(u)$ and its sample analogue $\hat{\boldsymbol{\alpha}}_3(u)$ defined in Definitions 4 and 5 of the article, respectively.

The following proposition provides basic properties of the trivariate measure which are similar to those of the bivariate measure presented in Proposition 2.

Proposition S1 *Let $\mathscr{C}_3$ be a set of all trivariate copulas. Suppose $\boldsymbol{\alpha}_{3,C_3}(u)$ is the measure $\boldsymbol{\alpha}_3(u)$ for the copula $C_3 \in \mathscr{C}_3$. Assume that $p_{3L} = C_3(u,u,u)$ and $p_{3U} = 1 - 3\bar{u} + C_3(\bar{u},\bar{u},1) + C_3(\bar{u},1,\bar{u}) + C_3(1,\bar{u},\bar{u}) - C_3(\bar{u},\bar{u},\bar{u})$. Let $C_{3P,jk\ell}$ be a permuted copula of C_3 defined by $C_{3P,jk\ell}(u_j,u_k,u_\ell) = C_3(u_1,u_2,u_3)$, where (j,k,ℓ) is a permutation of $\{1,2,3\}$. Define $\overline{C}_3$ by the survival copula associated with C_3. Then, for $0 < u \leq 0.5$, it follows that:*

(i) $-\infty \leq \boldsymbol{\alpha}_{3,C_3}(u) \leq \infty$ *for every* $C_3 \in \mathscr{C}_3$*; in particular,* $\boldsymbol{\alpha}_{3,C_3}(u) = -\infty$ *if* $p_{3U} = 0$ *and* $p_{3L} > 0$ *and* $\boldsymbol{\alpha}_{3,C_3}(u) = \infty$ *if* $p_{3L} = 0$ *and* $p_{3U} > 0$*;*
(ii) $\boldsymbol{\alpha}_{3,C_3}(u) = 0$ *if and only if* $p_{3L} = p_{3U}$*;*
(iii) for fixed p_{3U}*,* $\boldsymbol{\alpha}_{3,C_3}(u)$ *is monotonically non-increasing with respect to* p_{3L}*; similarly, for fixed* p_{3L}*,* $\boldsymbol{\alpha}_{3,C_3}(u)$ *is monotonically non-decreasing with respect to* p_{3U}*;*
(iv) $\boldsymbol{\alpha}_{3,C_3}(u) = -\boldsymbol{\alpha}_{3,\overline{C}_3}(u)$ *for every* $C_3 \in \mathscr{C}_3$*;*
(v) $\boldsymbol{\alpha}_{3,C_{3P,jk\ell}}(u) = \boldsymbol{\alpha}_{3,C_3}(u)$ *for any* $C_3 \in \mathscr{C}_3$*,* $j,k,\ell = 1,2,3$*,* $j \neq k$*,* $k \neq \ell$*,* $j \neq \ell$*;*
(vi) if $C_3 \in \mathscr{C}_3$ *and* $\{C_{3,n}\}_{n\in\mathbb{N}}$ *is a sequence of copulas such that* $C_{3,n} \to C_3$ *uniformly, then* $\boldsymbol{\alpha}_{3,C_{3,n}} \to \boldsymbol{\alpha}_{3,C_3}$*.*

As briefly mentioned in Section 9 of the article, the weak convergence to a Gaussian process holds for the sample analogue $\hat{\boldsymbol{\alpha}}_3$ of the trivariate measure defined in Definition 5. Details are given in the following theorem. The proof of this theorem is straightforward from that of Theorem 4 and therefore omitted.

Theorem S1 *Let $(U_{11},U_{21},U_{31}),\ldots,(U_{1n},U_{2n},U_{3n})$ be an iid sample from the trivariate copula $C_3(u_1,u_2,u_3)$. Assume*

$$\mathbb{A}_{3,n}(u) = \sqrt{n}\left\{\hat{\boldsymbol{\alpha}}_3(u) - \boldsymbol{\alpha}_3(u)\right\}, \quad 0 < u \leq 0.5.$$

Then, as $n \to \infty$, $\{\mathbb{A}_{3,n}(u) \,|\, 0 < u \leq 0.5\}$ converges weakly to a centered Gaussian process with covariance function

$$\begin{aligned}\sigma_3(u,v) &\equiv \mathbb{E}[\mathbb{A}_{3,n}(u)\mathbb{A}_{3,n}(v)] \\ &= \frac{C_3(u\vee v,u\vee v,u\vee v) + \overline{C}_3(\bar{u}\wedge\bar{v},\bar{u}\wedge\bar{v},\bar{u}\wedge\bar{v})}{C_3(u\vee v,u\vee v,u\vee v)\cdot\overline{C}_3(\bar{u}\wedge\bar{v},\bar{u}\wedge\bar{v},\bar{u}\wedge\bar{v})}.\end{aligned}$$

Inferential methods based on $\hat{\boldsymbol{\alpha}}_3(u)$ can be established by following the discussion in Section 5.2 and applying Theorem S1. For example, a $100(1-p)\%$ nonparametric asymptotic confidence interval for $\boldsymbol{\alpha}_3(u)$ can be established as

$$\hat{\boldsymbol{\alpha}}_3(u) - \frac{z_{p/2}\hat{\sigma}_3(u)}{\sqrt{n}} \leq \boldsymbol{\alpha}_3(u) \leq \hat{\boldsymbol{\alpha}}_3(u) + \frac{z_{p/2}\hat{\sigma}_3(u)}{\sqrt{n}},$$

where $\hat{\sigma}_3(u) = [\{T_{3L}(u) + T_{3U}(u)\}/\{T_{3L}(u)\cdot T_{3U}(u)\}]^{1/2}$ and $z_{p/2}$ is defined as in (9).
